# Autonomy undermined: how NHS institutional culture fails pregnant patients: Explaining the limits of autonomy in maternity care

**DOI:** 10.1093/medlaw/fwag027

**Published:** 2026-08-08

**Authors:** Mélinée Kazarian, Dani O’Connor

**Affiliations:** Law Department, School of Law and Politics, University of Cardiff, Cardiff CF103AX, United Kingdom; Bristol Medical School, University of Bristol, Bristol BS8 1UD, United Kingdom

**Keywords:** Autonomy, Healthcare Failure, Pregnancy, Medical Paternalism

## Abstract

Maternity care in the UK continues to confront profound and systemic failings, despite decades of legal and ethical commitments to patient-centred care. This article demonstrates that these failings are not simply operational or episodic but stem from an entrenched institutional culture of paternalism that routinely suppresses women’s autonomy. Drawing on major maternity inquiries alongside empirical studies documenting women’s experiences of pregnancy and childbirth, the paper illustrates how women’s voices are silenced, alternatives withheld, and concerns minimized—particularly for those from racialized or otherwise marginalized groups. These patterns reveal that autonomy, as currently conceived and practiced, remains largely procedural and illusory. The article argues that the dominant ‘individualistic’ model of autonomy cannot withstand the relational, emotional, and structural realities of pregnancy and childbirth, nor counter the gendered and hierarchical assumptions embedded within NHS practice. It proposes instead a shift towards a relational understanding of autonomy—one that recognizes vulnerability, prioritizes communication and trust, and situates decision-making within wider social and institutional contexts. Only by transforming the cultural foundations of maternity care can the promise of genuine autonomy and safe, respectful care be realized.

## A. Introduction

Despite significant advancements in legal and ethical frameworks surrounding patient care, maternity services in the UK remain plagued by systemic and recurring failures.^1^^,^^2^ These are not isolated incidents of mismanagement or individual negligence; rather, they reflect an entrenched institutional culture of paternalism, one that continues to obstruct women’s autonomy and compromise their quality of care during childbirth.^3^^,^^4^ This article argues that these failures must be understood not merely as instances of poor practice, but as the product of institutional cultures and structural conditions that undermine and, at times, bypass meaningful engagement with patient autonomy altogether. While maternity care is the focal case, the analysis also speaks to wider concerns about organizational culture and patient voice within the NHS. The formal appearance of informed consent frequently masks persistent patterns of institutional silencing and coercion.^5^ Women, especially those from racialized, socioeconomically disadvantaged, or otherwise marginalized backgrounds, are regularly denied meaningful agency over their own care,^6^ despite the landmark *Montgomery* ruling, which has been widely discussed in the literature as capable of strengthening patient autonomy.^7^ Inquiries into maternity care failings demonstrate that, in many cases, clinicians and institutions fail to engage with autonomy in any meaningful sense. Even where autonomy is formally invoked, it is reduced to a narrow, procedural construct—detached from the relational and institutional conditions in which decisions are actually formed. Deep-rooted assumptions about vulnerability and competence continue to shape professional behaviour, obstructing efforts to achieve respectful, responsive, and autonomous care.^8^ While legal and ethical frameworks purport to protect patient rights, they have proven insufficiently robust to challenge or transform the institutional conditions that enable these failures.^9^ First, that the most serious failures in maternity care often reflect not the misapplication of autonomy, but its absence in practice. Second, that even where autonomy is engaged, the prevailing model is understood too narrowly to capture the relational, institutional, and power-laden realities of decision-making in maternity care. As a result, it fails to provide an adequate framework for identifying, explaining, or addressing the systemic erosion of patient agency.

Through thematic analysis of key maternity inquiries—including Morecambe Bay,^10^ East Kent,^11^ Telford and Shrewsbury,^12^ Nottingham,^13^ and the ongoing Independent National Maternity Inquiry^14^—this article demonstrates how institutional structures systematically marginalize patient voices, undermine dignity, and contribute to preventable harm. These inquiries reveal strikingly similar patterns: at Morecambe Bay, avoidable deaths were linked to a ‘lethal mix’ of poor teamwork and denial; in East Kent, families were dismissed and blamed; at Telford and Shrewsbury, hundreds of cases showed patterns of coercion and neglect; and in Nottingham, women were pressured into unsafe births. These findings point to a culture dominated by professional hierarchy and disregard for informed consent—not isolated failures, but symptoms of a systemic problem embedded in the very fabric of maternity care. The analysis draws on a combination of deductive themes derived from autonomy, consent, and paternalism scholarship (including relational and feminist critiques).

The limitations of existing legal protections further compound this problem. While *Montgomery*^15^ appeared to mark a significant shift towards recognizing patient autonomy and informed consent,^16^ the recent *McCulloch*^17^ decision signals a worrying drift back towards clinical discretion.^18^ This jurisprudential regression—discussed in depth in Section B—is symptomatic of a wider failure: although bioethical principles—such as those of Beauchamp and Childress^19^—have championed patient-centred care, both law and ethics often fall short in practice.^20^ They remain too abstract, inconsistent, or procedurally weak to counteract entrenched hierarchies and cultural norms that sideline patients.^21^ The procedural translation of autonomy does not directly produce the most extreme failures documented in the inquiries. However, it creates a clinical and institutional environment in which autonomy is reduced to formality, thereby normalizing practices that fall short of genuine engagement and allowing more serious disregard for patient agency to persist unchecked.

This article offers a distinctive and timely contribution by showing that genuine autonomy in maternity care is unattainable unless we confront not only the conceptual limits of prevailing legal and ethical frameworks, but also the entrenched institutional cultures that shape their application. The dominant model of autonomy—which we argue tends to be individualistic, procedural, and context-blind—is ill-suited to the emotional, clinical, and structural complexity of maternity care.^22^ Accordingly, this article highlights how reliance on this narrow conception of autonomy contributes to paternalism and undermines patient agency, particularly in the maternity context.

The central purpose of this article is diagnostic: it exposes the shortcomings of traditional autonomy and shows, through doctrine, professional guidance and inquiry evidence, how patient voices are systematically undermined in maternity care. It also advances the conceptual basis for a relational understanding of autonomy, which conceives autonomy not as isolated individual choice but as socially embedded and shaped by relationships, power structures and conditions of support.^23^ A companion paper develops the constructive response in operational terms, translating relational autonomy into a practical framework for clinical communication, organizational priorities, and institutional reform.

Following the introduction, this article proceeds in four parts. Section B sets out the analytical framework, drawing on consent doctrine, professional guidance and ethical theory to show how autonomy is formally protected yet structurally constrained in maternity care, with particular attention to *Montgomery*, *McCulloch* and the procedural limits of an individualistic model. Section C then turns to the evidence from NHS maternity inquiries, using thematic analysis to demonstrate how institutional cultures of hierarchy, denial and gendered power routinely silence women, distort consent processes and contribute to preventable harm. Building on these doctrinal and empirical findings, section D develops the conceptual case for a relational and context-sensitive understanding of autonomy as a better fit for maternity care’s communicative and power-laden realities, while leaving the detailed operationalization of this framework to the companion paper. Section E concludes by synthesizing the argument and identifying the implications for health law, bioethics and maternity governance.

## B. Analytical framework

This section sets out the legal and ethical framework governing informed consent in medical treatment, with a particular focus on how autonomy is conceptualized and operationalized in maternity care. It first examines the shift towards patient-centred decision-making in *Montgomery*, before considering the subsequent recalibration of that approach in *McCulloch*, which reintroduces professional discretion in the discussion of treatment alternatives. The section then turns to professional and ethical guidance to assess whether these normative commitments to autonomy are realized in practice. The analysis demonstrates that although law and ethics formally endorse respect for patient autonomy, they rely on a procedural and individualistic model that is ill-suited to the realities of maternity care. This framework provides the foundation for the argument developed in the remainder of the article: that the apparent protection of autonomy masks its systematic erosion in practice, a failure that becomes visible through the findings of maternity inquiries examined later in the paper.

### I. *Montgomery:* a turning point for patient empowerment?

In this case, the appellant, Mrs Montgomery, sought damages against Lanarkshire Health Board alleging negligence on the part of Dr McLellan, the obstetrician responsible for her care during pregnancy and labour. Her son was born with cerebral palsy, a severe disability, as a result of shoulder dystocia during delivery.^24^ Nadine Montgomery, who is around five feet tall and diabetic, fell within a higher-risk category: diabetic women are more likely to have larger babies, and the risk of shoulder dystocia in vaginal delivery is approximately 9–10 per cent.^25^ During her care, she expressed concerns to Dr McLellan that her baby might be too large for a safe vaginal delivery, though she had not asked about ‘exact risks’.^26^ Nevertheless, she was never informed of the risk of shoulder dystocia nor offered the option of a caesarean section, which would have averted this outcome.^27^ Dr McLellan later acknowledged that, had Mrs Montgomery been told of the risk, she would likely have opted for a caesarean section—an option the treating doctor did not believe to be in her best interest.^28^ While it was accepted that shoulder dystocia can lead to serious complications for both mother and child, it was also noted that in her particular case the risk of cerebral palsy was low, at around 0.1 per cent.^29^ The Outer House of the Court of Session in Scotland dismissed Mrs Montgomery’s claim on the grounds that no duty of care had arisen in the circumstances. It reasoned that she had not shown she would have chosen a caesarean section if she had been warned of the relevant risks. The Lord Ordinary applied the prevailing legal precedent at the time, *Sidaway*, which held that a doctor’s failure to warn a patient of treatment risks would only amount to a breach of duty if it failed the *Bolam* test—namely, whether the omission was supported by a responsible body of medical opinion that could not be considered irrational (as clarified in *Bolitho*).^30^ The Lord Ordinary concluded that the *Bolam* test had not been breached. In his view, although the risk of shoulder dystocia was notable, it did not, in itself, necessitate a warning, as it was usually managed with ‘simple procedures’ and the likelihood of serious harm to the baby was ‘tiny’.^31^ Consistent with *Sidaway*, he also noted that a doctor is required to answer if a patient enquires about specific risks but found that Mrs Montgomery had not made such inquiries. The Inner House of the Court of Session rejected Mrs Montgomery’s reclaiming motion and affirmed the Lord Ordinary’s decision.^32^

The UK Supreme Court unanimously overturned the decisions of the Scottish Courts, allowed Mrs Montgomery’s appeal and overruled *Sidaway.* Lords Kerr and Reed held that the doctor-patient relationship model underlying *Sidaway* no longer reflected modern reality. Patients today are seen as capable adults, not passive recipients of medical advice, and the law must support their autonomy and right to make informed decisions about their own care.^33^ Doctors have a legal duty to ensure patients are made aware of material risks and reasonable alternatives to treatment. Since this duty is grounded in respect for patient autonomy—not medical expertise—the *Bolam* test is unsuitable in this context.^34^

An adult of sound mind is entitled to decide what treatments to undergo, and doctors must facilitate that choice through dialogue, not paternalism. A risk is ‘material’ if a reasonable patient would likely consider it significant, or if the doctor should reasonably know that their particular patient would.^35^

In this case, Dr McLellan should have disclosed the risk of shoulder dystocia. The lower courts focused too narrowly on the small risk of serious injury to the baby, rather than on the emergency nature of shoulder dystocia itself. Crucially, Dr McLellan acknowledged that Mrs Montgomery would likely have opted for a caesarean if informed—indeed, she withheld the information for that reason.^36^ The only reasonable conclusion was that, if properly advised, Mrs Montgomery would have chosen a caesarean.^37^

Lady Hale emphasized that treatment decisions cannot be separated from their alternatives—especially in pregnancy, where both maternal and foetal risks are at stake. Once a doctor’s judgment involves non-medical values, the *Bolam* test no longer applies. A competent patient is entitled to information that enables meaningful participation in the decision-making process.^38^

In her judgment, Lady Hale observed that Dr McLellan would explain to women requesting a caesarean section ‘why it may not be in the mother’s best interest’ and that she considered ‘it’s not in the maternal interests for women to have caesarean sections’.^39^ It is clear that Dr McLellan’s decision was not purely medically motivated. Rather, it reflected the belief that vaginal delivery was morally preferable to a caesarean section, a belief strong enough to justify withholding information that Mrs Montgomery needed in order to make a free choice. As Lady Hale emphasized, the clinician’s duty is to ensure the patient is aware of material risks and reasonable alternatives so she can make that decision for herself.^40^ As Lady Hale powerfully stated, ‘gone are the days when it was thought that, on becoming pregnant, a woman lost, not only her capacity, but also her right to act as a genuinely autonomous human being’.^41^ Her judgment underlined that choices about childbirth are not simply clinical but deeply personal and value-laden and that paternalistic reasoning of the kind displayed by Dr McLellan in the *Montgomery* case is incompatible with genuine respect for autonomy.

The impact of stereotypical assumptions in maternity care is clearly illustrated in this case by the doctor’s belief that vaginal delivery was in the mother’s best interests. Notably, these assumptions were made by a female clinician, underscoring that the issue is not one of individual bias but a reflection of systemic attitudes within the healthcare profession.^42^ This approach often sidelines women’s voices, replacing informed, supported decision-making with what clinicians—regardless of gender—perceive to be the ‘right’ course of treatment. As a result, maternity care can become a space in which women’s preferences and decision-making authority are undermined, despite the formal legal commitment to autonomy.^43^ While the doctrine intends to protect patient autonomy, it fails to do enough to balance bias.^44^ In terms of reproduction, women are not provided with the balanced and comprehensive information required to promote their autonomy.^45^ A need exists for a broader framework to be developed which would support women through the decision-making process concerning their reproductive rights. While Montgomery represents a significant advance in the legal protection of patient autonomy, the case also illustrates the limits of a framework centred primarily on disclosure. The judgment addresses the information patients receive, but is less equipped to engage with the broader institutional, relational and cultural factors that shape how choices are presented, understood and acted upon in practice. As the maternity inquiries discussed later demonstrate, failures of autonomy often arise not simply from inadequate disclosure, but from the wider conditions within which decision-making takes place.

### II. Post-*montgomery* case law: *McCulloch* and the retreat to professional discretion

In March 2012, Mr McCulloch was admitted to Forth Valley Royal Hospital with chest pains, nausea and vomiting. Consultant cardiologist Dr Labinjoh reviewed his echocardiogram and judged that his symptoms did not match a typical diagnosis of pericarditis. His condition initially improved, and he was discharged, but he was readmitted shortly after with recurring chest pain. On review of a further echocardiogram, Dr Labinjoh noted that Mr McCulloch appeared better, denied chest pain, and therefore she did not consider it appropriate to prescribe non-steroidal anti-inflammatory drugs (NSAIDs) such as ibuprofen. He was discharged on 6 April, but the following day he suffered a fatal cardiac arrest at home.

Mr McCulloch’s widow and family alleged negligence, arguing that Dr Labinjoh had breached her duty of care by failing to inform him that NSAIDs were a potential treatment option and that he would have taken them if advised, preventing his death. Expert evidence showed that while some doctors would have prescribed NSAIDs, a responsible body of medical opinion supported Dr Labinjoh’s decision not to do so, given the absence of pain and no clear diagnosis of pericarditis. Both the Lord Ordinary and the Inner House found that Dr Labinjoh was not negligent in her approach. The family appealed this decision to the Supreme Court, which dismissed the appeal unanimously. The Supreme Court held that Dr Labinjoh had not breached her duty of care in deciding against prescribing NSAIDs, as this decision was supported by a responsible body of medical opinion.

In *McCulloch*, the UK Supreme Court prioritized medical professional opinion over patient autonomy,^46^ particularly regarding the discussion of alternative treatments, a key point of divergence from *Montgomery*. The ruling effectively reintroduces the *Bolam* test into an area previously governed by a patient-focused standard, weakening the emphasis on informed patient choice.

The appellants in *McCulloch* had endorsed the two-stage test proposed by the Court of Appeal in *Duce*,^47^ whereby the first stage (what risks or alternatives should be disclosed) would be assessed under *Bolam*, and the second stage (how much information must be given) under *Montgomery*.^48^ The Supreme Court, however, rejected this approach, reinforcing a more deferential stance towards clinical judgment.^49^ The consequences for patient care, especially in maternity services, are significant. As Hobson observes, had the *McCulloch* reasoning been applied to the facts in *Montgomery*, where the claimant was not informed of the option of a caesarean section, she likely would have lost her claim. This highlights how the shift in legal reasoning could endanger patient rights.^50^

The case also introduces practical ambiguity.^51^ While the *Montgomery* test continues to govern risk disclosure, the *Bolam* test now applies to the discussion of alternatives. This dual standard creates uncertainty in clinical practice: do healthcare professionals clearly understand which test governs which aspect of the consent process and how to navigate between them? As such, concerns arise about whether the profession is aware of these nuances and how the legal framework aligns with ethical guidance on informed consent, decision-making and patient communication.

The broader argument here is not that *McCulloch* creates paternalism in maternity care, but that it may reinforce existing paternalistic practices by restoring professional control over which alternatives are presented to patients. It conveys a troubling message to healthcare providers: so long as their judgment aligns with peer-approved standards, they can decide what is ‘best’ for the patient without facing liability.^52^ This stance is particularly concerning in light of official inquiries, which have documented repeated failures to listen to women or offer alternative options, failures that in some cases led to preventable deaths of mothers or babies, which will be shown in Section C.

While the inquiries document a wide range of systemic failings—including unsafe staffing levels, poor escalation practices, organizational dysfunction and cultures of denial—they also reveal a recurring pattern directly relevant to the legal issues raised in *McCulloch*. Alternatives were not consistently presented as genuine options, women were steered towards preferred clinical pathways, and requests for different modes of birth were often treated as unreasonable or disruptive. *McCulloch* did not create these practices. However, by restoring professional discretion over which treatment alternatives must be discussed, it risks providing legal shelter for decision-making cultures in which patient preferences are marginalized. The concern is therefore not that *McCulloch* causes the failures documented in the inquiries, but that it leaves the law less able to challenge or remedy them.

Under *McCulloch*, such conduct might not trigger legal responsibility, thereby enabling dangerous practices to persist.

### III. The limits of the prevailing model of autonomy

Current UK guidance on pregnancy and childbirth formally places significant emphasis on patient autonomy, informed decision-making, and respectful care across the antenatal, intrapartum, and postnatal continuum.^53^

Respect for autonomy is commonly understood as requiring clinicians to listen to patients’ concerns, engage in meaningful dialogue, and support informed decision-making rather than merely securing consent. This includes enabling patients to make choices that reflect their own values and priorities, even where those choices diverge from professional recommendations. The National Institute for Health and Care Excellence (NICE) promotes shared decision-making as a core ethical commitment, presenting maternity care as a collaborative process in which multiple clinically appropriate options should be discussed and patient preferences taken seriously.^54^ This emphasizes individualized care, responsiveness to values, and respect for choice.

However, while these ethical commitments are clearly articulated, they remain largely aspirational. In practice, maternity care is shaped by a persistent dual-focus on the pregnant person and the foetus, a framing that frequently reopens space for professional discretion to shape or constrain the choices presented to pregnant patients.^55^ Ethical guidance formally rejects paternalism, yet simultaneously tolerates it through vague standards that permit clinicians to determine which options are sufficiently ‘reasonable’ to warrant discussion. As the following sections demonstrate, the legal doctrine of consent has both challenged and reinforced these dynamics, resulting in a framework that appears autonomy-protective in theory while enabling its erosion in practice.

Although recent case law and professional guidance purport to embrace autonomy, consent in maternity care frequently collapses into a procedural or bureaucratic exercise. Rather than facilitating genuine shared decision-making, it often operates as a ‘tick-box’ mechanism aimed at legal defensibility rather than ethical engagement.^56^ This proceduralization of consent undermines its ethical purpose: instead of empowering patients through dialogue, it reduces autonomy to the formal acquisition of agreement, detached from meaningful understanding or deliberation.^57^

The most recent GMC guidance on consent explicitly frames clinicians as responsible for ‘supported decision-making’ and requires discussions to be tailored to the individual patient in order to satisfy the test of materiality. *Decision Making and Consent* repeatedly emphasizes that the doctor–patient relationship should function as a partnership grounded in openness, trust, and effective communication. Paragraphs 17 and 18 are particularly significant, instructing clinicians to explore what matters to patients, including their values, fears, and expectations, so that decisions reflect the patient’s own priorities rather than professional assumptions.^58^

Yet despite this strong ethical rhetoric, translation into practice remains limited. A central weakness lies in the unresolved ethical and legal question of what constitutes a ‘reasonable’ option that must be presented to the patient. This standard introduces an objective threshold that closely resembles *Bolam*-style reasoning, permitting clinicians to filter information through professional norms before it reaches the patient. As a result, the ethical ideal of supported decision-making is quietly hollowed out. Consent may be formally obtained, but the underlying ethical commitment to respect for autonomy is compromised. The persistent gap between contrived consent in practice and bona fide consent in principle remains unresolved. The persistent gap between formally valid consent and genuinely informed and supported decision-making remains unresolved.^59^^,^^60^

These shortcomings are exacerbated by the structural realities of the NHS. Time pressures, staff shortages, risk-averse institutional cultures and entrenched professional hierarchies significantly constrain opportunities for meaningful conversation.^61^ In this context, ethical obligations that depend on dialogue, reflection and trust are systematically undermined. Consent is secured, but autonomy is not realized.

The problem, however, is not merely practical. It is also conceptual. The prevailing legal and ethical framework presumes an independent decision-maker capable of exercising autonomy in isolation from their social, emotional and institutional context.^62^ In maternity care—more than in any other area of medicine—this abstraction is particularly ill-suited.^63^ Decisions about childbirth are inherently relational, emotionally charged, and shaped by vulnerability, power imbalance and competing narratives of risk.^64^ The failure of autonomy in maternity care does not stem solely from the traditional concept of autonomy on which law and ethics rely. Rather, the inquiry evidence suggests a more fundamental problem: the conditions required for any meaningful exercise of autonomy are frequently absent in practice. However, the limitations of the prevailing model remain significant, as it fails to capture or respond to these institutional and relational dynamics.

At the heart of these failures lies a deficit of genuine communication. While legal and ethical guidance repeatedly emphasizes dialogue, in practice such dialogue is often one-sided. Clinicians dominate discussions, women’s concerns are marginalized, and their preferences are reframed or dismissed. This dynamic reflects the persistence of the ‘doctor knows best’ mentality, sustained by deeply embedded stereotypes about women’s capacity for rational decision-making. Emotional responses are frequently discounted as irrational or unreliable, reinforcing gendered assumptions that undermine women’s credibility as decision-makers. As Bell and others observe, male authority continues to be linguistically associated with ‘order’ and ‘management’, while female voices are diminished through association with emotion and instability.^65^ In maternity care, these stereotypes actively legitimize medical paternalism by recasting decisions made for women as protective rather than coercive.

### IV. Relational autonomy: a contextual approach

Relational autonomy, developed within feminist bioethics and political theory, challenges the assumption that individuals are self-sufficient, independent decision-makers. Instead, it understands autonomy as socially constituted: shaped by relationships, power structures, and the conditions in which individuals form and express their preferences.^66^ On this account, autonomy is not simply the capacity to make a choice at a particular moment, but a process dependent on recognition, support, and meaningful engagement over time.

This differs fundamentally from the current model that underpins legal doctrine. Whereas the prevailing framework locates autonomy at the point of consent—focusing on disclosure and decision—relational autonomy locates it in the conditions under which decisions are formed. It asks not only whether a choice was made, but how that choice came to be shaped: whether the individual was heard, whether alternatives were meaningfully available, and whether power imbalances distorted the decision-making process. In this sense, relational autonomy reconceptualizes autonomy as a socially embedded practice rather than a discrete act of individual will.

This paper does not propose a new legal test. Rather, it argues for a more nuanced ethical understanding of autonomy in practice, one that is capable of bridging the persistent gap between doctrinal commitments and lived experience. A relational approach to autonomy may offer such a framework.^67^ By shifting attention away from abstract individualism, relational autonomy recognizes the realities of women’s lives, including family responsibilities, social positioning, dependency, vulnerability, and the emotional dimensions of decision-making. Importantly, it does not weaken autonomy but reframes it in a way that better reflects how decisions are actually made in maternity contexts. Recognizing the relational dimensions of decision-making does not justify overriding women’s choices in the name of their welfare. Rather, it highlights the social, communicative and institutional conditions necessary for meaningful self-determination, while preserving the individual’s authority to make decisions about their own care.

By foregrounding relational autonomy, it becomes possible to promote balance in the doctor-patient relationship and to re-centre communication as the ethical core of informed consent. The persistence of autonomy failures in maternity care exposes a fundamental flaw in the prevailing model: it places the formal burden of decision-making on women, while leaving intact the professional and institutional structures through which medical paternalism operates. Autonomy is therefore affirmed in principle but routinely undermined in practice. This reflects a deeper conceptual limitation: autonomy is mislocated as a discrete moment of consent rather than understood as a process formed over time. By reducing it to disclosure and agreement, the model obscures how decisions are shaped by communication, trust, institutional pressures and power dynamics, with the result that autonomy is recognized in form but not realized in substance. The following section demonstrates how these failures manifest in clinical reality, drawing on evidence from major NHS maternity inquiries to show how paternalism and the marginalization of women’s voices have become embedded within maternity care.

## C. System failure and gendered power: how paternalism shapes NHS maternity care

Before turning to the thematic analysis, it is important to clarify the nature of the failures documented in these inquiries. In many instances, the conduct described reflects not the application of a flawed or overly individualistic model of autonomy, but a more fundamental breakdown in which autonomy is not meaningfully engaged at all. Women were ignored, dismissed, or actively overruled in ways that cannot plausibly be explained by any coherent account of informed decision-making. This section therefore does not attribute these harms solely to the limitations of the dominant autonomy framework. Instead, it shows how institutional culture, hierarchy and epistemic injustice create conditions in which the basic requirements of autonomy—listening, recognition, and respect for the patient as a decision-maker—are routinely absent. Nonetheless, this absence does not render the critique of the prevailing model redundant. On the contrary, it exposes its limitations. Even where autonomy is formally invoked, the current framework lacks the conceptual resources to account for the relational and institutional conditions that shape whether autonomy can be meaningfully exercised in practice.

### I. Silencing women and disregarding consent

One of the clearest and most consistent failings identified across the inquiries is the routine dismissal of women’s voices. At the most basic level, women reported being ignored when they expressed pain, distress, or concern. In the East Kent Report, for example, a woman described her experience of spinal and epidural analgesia during surgery: ‘they didn’t listen … they carried on, obviously, to cut me open. I could feel it all’.^68^ Her testimony captures both the physical trauma and the indignity of being unheard, as clinicians proceeded regardless of her protests.

Failures to listen were not confined to pain management but extended to women’s preferences for labour and delivery. Requests for caesarean sections were frequently disregarded, either without meaningful discussion or on the basis of clinicians’ judgments about what was considered to be in the patient’s best interests. The inquiries noted repeated instances where alternatives were not properly explained, leaving women unable to exercise meaningful choice. In several cases highlighted by the Ockenden Review, mothers reported that their options were never discussed with them, effectively excluding them from decisions about their own care.^69^

These examples show that the principle of informed consent, central to both law and ethical guidance, was consistently undermined in practice. Consent was treated as a box-ticking exercise, with information presented selectively or not at all.^70^ Instead of a collaborative process, women encountered unilateral decision-making, where clinical authority dictated the course of action and patient preferences were overridden. From an autonomy perspective, these practices are deeply problematic. While the current legal model assumes that once information is provided, the patient can make a rational, independent choice, the inquiry evidence demonstrates something more fundamental: women were often denied the basic conditions required for any meaningful exercise of autonomy in the first place. In this way, their autonomy was not simply overlooked but actively obstructed. Women were positioned as passive recipients of care rather than as agents with the right to determine what happened to their bodies.

The silencing of women and the disregard for consent illustrate the hollowness of autonomy when reduced to a procedural formality, and, in many cases, its complete absence in practice. Genuine respect for autonomy requires not only the disclosure of risks and options but also a willingness to listen, engage and respect the patient’s values. Where these conditions were absent, as the inquiries repeatedly demonstrate, women’s autonomy was rendered illusory.

### II. Coercion, blame, and a culture of intimidation

Alongside failures to listen, the inquiries uncovered a pervasive culture of coercion and blame that further undermined women’s autonomy. Families who sought to question clinical decisions were often met not with openness, but with hostility or dismissal. At East Kent, parents who raised safety concerns described being treated as ‘difficult’, their worries brushed aside rather than engaged with. In this environment, women and families learned that voicing concerns could invite judgement or rejection rather than support.

In the most extreme cases, bereaved mothers were directly subjected to blame or platitudes that trivialized their loss. One woman, whose baby died, recalled being told: ‘It’s God’s will; God only takes the babies that he wants to take’.^71^ Such statements reveal not only a profound lack of compassion, but also a refusal to acknowledge professional responsibility. Similarly, another mother was visited by a staff member after a critical error had been missed at birth. Rather than asking about her baby’s wellbeing or offering an apology, the staff member sought to deflect responsibility by saying: ‘You do remember I was handing over, don’t you?’^72^ These accounts highlight the ways in which paternalistic authority can morph into defensiveness, leaving families isolated in their grief.

The culture of intimidation extended to staff themselves. At East Kent, midwives and junior doctors described being shouted at, humiliated in front of patients and excluded by senior colleagues. An anonymous staff letter in 2014 painted a vivid picture of the working environment: ‘I dread going to work with certain people… Management and those with authority are not approachable, there is a blame culture, a just get on with it and shut up attitude… At times the unit is [an] awful place to be’.^73^ Such testimonies were corroborated by a review in which over half of staff reported experiences of bullying and intimidation, including racism, sarcasm and fear of retaliation.

This culture of coercion and blame has clear consequences for autonomy. When patients fear being labelled ‘difficult’, they are less likely to question or resist decisions made on their behalf. When staff fear retribution, they are less likely to advocate for patients or challenge unsafe practices. In both cases, authority is preserved not through collaboration but through intimidation, silencing the very voices that might otherwise hold the system accountable.

From an autonomy perspective, coercion corrodes the possibility of genuine choice. Consent obtained in a context of fear or blame cannot be considered meaningful because the conditions necessary for free and informed decision-making are absent. The inquiries suggest that coercive and defensive organizational cultures were not incidental but systemic. These cultures did not simply influence individual decisions; they undermined the conditions required for meaningful autonomy to be exercised at all.

### III. Absence of compassion and professional accountability

A further recurrent pattern across the inquiries was the absence of compassion and a failure of professional accountability. Women repeatedly described feeling ignored, dismissed, or treated without kindness during some of the most vulnerable moments of their lives. The Ockenden Review concluded starkly that kindness and compassion were too often lacking even in cases where outcomes were tragic, and families were left grieving.^74^ For women in labour or postnatal care, the absence of basic empathy compounded trauma and eroded trust in those responsible for their safety.

Examples illustrate the depth of this problem. At East Kent, one mother described undergoing surgery while crying out in pain, yet her distress was disregarded.^75^ Others reported being left alone or made to feel like a burden when they sought support. Such experiences made women feel not only uncared for but dehumanized, their suffering treated as secondary to clinical process. Compassion is not merely an optional virtue in maternity care; it plays an important role in creating the conditions for meaningful dialogue, trust and respect for patient values. Where those conditions are absent, opportunities for autonomous decision-making may be significantly diminished.

Alongside the lack of compassion, inquiries highlighted systemic failures of professionalism. At Morecambe Bay, team dysfunction between obstetricians and midwives led to unsafe practices, with neither group taking responsibility for escalating risks.^76^ The East Kent inquiry described similar breakdowns in communication and accountability, with ‘challenging personalities’ and cliquish behaviour undermining patient safety.^77^ One external assessor went so far as to call the unit’s culture ‘the worst [they] had ever seen’.^78^ In practice, this meant that serious incidents were neither properly reported nor investigated, and opportunities to learn from mistakes were consistently lost.

Professionalism and compassion matter because they help create the relational conditions within which meaningful decision-making can occur. Where communication breaks down, empathy is absent, and responsibility is evaded, women may be denied the engagement and recognition necessary to participate fully in decisions about their care. These failures are not reducible to problems of consent alone; they reflect broader organizational conditions that undermine trust, dialogue and patient agency. In this sense, compassion and accountability are not merely matters of professional conduct, but important components of the environment within which autonomy can be realized in practice.

### IV. Institutional and regulatory complacency

The inquiries also exposed the failures of both Trust-level leadership and external regulators to confront unsafe practices. At East Kent, senior managers were repeatedly made aware of bullying, poor teamwork and patient safety risks, yet chose to prioritize reputation management over reform. Action plans were routinely drafted but not implemented; staff who attempted to challenge poor practice were marginalized; and the Board consistently underestimated the scale of harm. As one account put it, ‘If there is one thing East Kent can do it’s write an action plan’,^79^ a damning indictment of a culture that valued appearance over substance.

At the regulatory level, oversight was equally ineffective. Despite the involvement of multiple agencies, systemic failings persisted unchecked for years. The East Kent inquiry observed that the ‘plethora of regulators’ created a diffusion of responsibility, allowing the Trust to deflect criticism rather than confront its shortcomings.^80^ Regulators themselves acknowledged the difficulty of penetrating the pervasive culture of denial, which extended from ward staff to board level. As the report found: ‘The task of regulators was made more difficult by the extent to which problems were denied; this denial ran right through the Trust, from clinical staff to Trust Board level.’^81^ The failure to properly investigate incidents or hold individuals accountable illustrated the system’s inability to secure meaningful change.

These institutional and regulatory deficiencies confirm that the problems in maternity care cannot be explained away as the result of a few negligent practitioners. Rather, they reflect deeply embedded organizational cultures shaped by paternalism, gendered assumptions and hierarchies of authority.^82^ Understanding these failures therefore requires looking beyond individual incidents and formal governance structures to the underlying assumptions, power dynamics and patterns of credibility that determine whose voices are heard, trusted and acted upon within maternity care.

### V. The epistemic foundations of paternalism in maternity care

The preceding sections have shown how autonomy is frequently absent in practice and inadequately captured by the prevailing model. This section examines the epistemic foundations of paternalism in maternity care, demonstrating why these failures persist despite clear legal and ethical commitments to autonomy. The argument advanced here is that paternalism is sustained not only by institutional structures, but by deeply embedded assumptions about pregnant women’s credibility, rationality and role. These epistemic dynamics shape how women’s voices are received, interpreted and, in many cases, dismissed. Pregnancy amplifies these dynamics in four key ways:

#### a. Stereotypes of irrationality and ‘baby brain’

Pregnant women are often stereotyped as overly emotional, irrational, or forgetful—a stereotype colloquially referred to as ‘baby brain’.^83^ Crawley and others note that such stereotypes portray women as ‘illogical, irrational, and forgetful’ during pregnancy,^84^ while Choi observes that intelligence and competence are positioned as diametrically opposed to the hallmarks of femininity.^85^ Longhurst argues that these stereotypes, underpinned by benevolent sexism and protective paternalism, cast pregnant women as helpless and in need of unsolicited advice. As she explains, ‘Pregnant women—their disorderly bodies and minds—[are] widely considered to be in need of a great deal of advice.’^86^ Such attitudes legitimize the restriction of women’s role in decision-making and normalize substituted rather than shared decision-making, where professionals act on behalf of patients rather than with them. These stereotypes operate as credibility deficits, systematically undermining women’s status as knowers in clinical encounters and diminishing the weight afforded to their accounts of pain, risk, preference and experience. In Fricker’s terms, this constitutes a form of testimonial injustice, whereby prejudice distorts the credibility granted to a speaker. ^87^

#### b. Foetus-centric care and the presumption of maternal incompetence

Unlike other medical contexts where patients can refuse treatment against medical advice, obstetric decision-making is anomalous in the weight it places on foetal welfare. Abrams describes women’s autonomy in this context as an ‘illusion’ with childbirth treated as an exception to the usual principle of bodily integrity.^88^ This is reflected in cases such as *MB (Caesarean Section)*,^89^ where a woman with needle phobia was treated as lacking capacity because her refusal of a caesarean section was judged irrational, and in the St George’s Healthcare litigation, where a competent woman’s refusal was overridden in the name of protecting the foetus.^90^ These examples demonstrate the tensions that can arise between formal commitments to autonomy and concerns about foetal welfare within obstetric decision-making.

This tendency is reinforced by the ambiguous legal position of the foetus. In principle, the law is clear that the foetus has no legal personality until it has a separate existence^91^ and does not benefit from Article 2 ECHR protection.^92^ Yet, the foetus is not treated as a legal ‘nothing’. Abortion remains criminalized under sections 58 and 59 of the Offences Against the Person Act 1861, subject to the statutory defences introduced by the Abortion Act 1967, and the Human Fertilisation and Embryology Acts 1990 and 2008 recognize a special status for embryos and foetuses. In practice, particularly where the foetus is viable or delivery is considered safe, concerns about foetal welfare may exert significant influence over clinical decision-making, creating pressure towards outcomes that can constrain maternal choice. Wood-Goldbeck notes that in no other field of healthcare are paternalistic practices so prevalent, even as patients increasingly reject them,^93^ and as Annas argues, pregnant women risk being treated as ‘foetal containers, non-persons without rights to bodily integrity’.^94^ The effect is that the foetus’s perceived interests often override the woman’s expressed preferences, creating what Cahill terms a uniquely gendered limitation on autonomy: ‘maternal autonomy’.^95^

#### c. Institutionalized stereotypes in practice

Qualitative research reinforces how these stereotypes shape clinical encounters. Yuill and others found that while healthcare professionals often believed they were offering informed choice, many women experienced this as superficial or illusory.^96^ Decisions were presented as procedural steps, with foetal wellbeing dominating interactions. Khan and others demonstrate that ethnic minority women in particular were subjected to pressured consent for high-intervention procedures, often delivered through fear-based language that made refusal difficult.^97^ MacLellan and others found that Black, Asian, and Minority Ethnic women in the UK frequently experienced maternity care as technocratic and impersonal, describing themselves as being ‘processed rather than cared for’.^98^

National surveillance data further confirm the systemic nature of these inequities.^99^ The MBRRACE-UK report continues to show stark and persistent racial disparities: Black women are 3.7 times more likely to die in pregnancy or up to six weeks postpartum than white women; women of mixed ethnicity are 1.8 times more likely; and Asian women 1.6 times more likely.^100^ These disparities have persisted despite sustained attention and cannot be fully explained by socioeconomic or clinical factors alone. Taken alongside qualitative evidence of differential treatment and communication, they raise serious concerns about the role of structural and institutional racism within maternity care.

Cultural and communication barriers further undermine genuine autonomy. The persistent lack of qualified interpreters, insufficient culturally competent communication, and limited accommodation of religious preferences, such as modesty considerations, choice of birth partner, or requests for female clinicians, restrict women’s ability to meaningfully participate in decision-making.^101^ Under the Equality Act 2010, a failure to provide culturally appropriate communication or to ensure equitable service provision may constitute indirect racial discrimination. These findings confirm that paternalism is not simply the product of individual prejudice but is institutionalized and racialized, disproportionately silencing marginalized women.

#### d. Broader patterns of maternal discrimination

The stereotyping of pregnant women extends beyond the clinic into wider social and economic structures. Research by the Equality and Human Rights Commission^102^ found that half of all mothers surveyed reported a negative impact on job opportunities, status, or security owing to maternity discrimination. Villarmea and Kelly add that epistemic stereotypes about women in labour reinforce the assumption that labouring women cannot meet the ideal of the rational agent.^103^ In practice, ‘supported decision-making’ often collapsed into clinicians ‘consenting the patient’—listing options and risks, then inviting a signature, rather than engaging in genuine shared dialogue. Autonomy is therefore not simply limited but unevenly realized in practice, functioning most effectively for those who are able to conform to the model’s expectation of the self-sufficient and socially empowered patient.

These dynamics demonstrate that paternalism in maternity care is not merely a product of individual behaviour or isolated institutional failure. It is epistemically embedded, sustained by assumptions about whose knowledge counts and whose decisions are considered credible. This helps to explain why autonomy is so often absent in practice: not simply because it is poorly implemented, but because the conditions necessary for recognizing women as autonomous agents are systematically undermined. Any attempt to reform maternity care must therefore address not only legal standards and institutional processes, but the deeper epistemic structures that shape clinical judgment and patient interaction. In this context, a relational understanding of autonomy becomes essential. By recognizing autonomy as dependent on conditions of trust, recognition and communicative engagement, a relational approach provides a framework capable of identifying and responding to the epistemic injustices that undermine women’s agency in practice. It therefore offers a more adequate account of how autonomy is enabled, constrained and sometimes foreclosed within maternity care.

## D. Beyond individualism: towards a relational model of autonomy

The evidence from maternity inquiries and the wider literature paints a consistent and troubling picture. The four themes of systemic failure: silencing women and disregarding consent; coercion and blame; absence of compassion and professionalism; and institutional and regulatory complacency, demonstrate how autonomy is routinely undermined in practice. Women were not only excluded from decisions about their own care but often actively overruled, intimidated, or dismissed.

The epistemic analysis reveals why these failings are so persistent. Stereotypes of irrationality and ‘baby brain’ diminish women’s credibility as decision-makers. Protective paternalism and foetus-centric care legitimize the substitution of professional judgment for maternal choice. Structural inequities and institutionalized biases, particularly against marginalized women, ensure that patient voices are systematically sidelined. These cultural logics underpin and reproduce the very patterns of failure identified in the inquiries, confirming that paternalism in maternity care is not incidental but embedded.

These findings suggest that the central challenge in maternity care is not simply the protection of autonomous choice at discrete moments of decision-making. Rather, it is the creation and maintenance of the conditions under which meaningful autonomy can be exercised at all. The inquiry evidence demonstrates that those conditions are relational, communicative and institutional in nature. Women require not only information, but recognition, trust, responsiveness and opportunities for genuine participation in decisions about their care. These observations point towards the need for a broader understanding of autonomy, one that can account for the social and structural contexts within which decisions are formed. It is to such an account that the discussion now turns.

### I. Why the current model fails in maternity care

The prevailing legal framework assumes an independent decision-maker who operates outside of social or institutional contexts.^104^ In maternity care, however, decisions about pregnancy and childbirth are necessarily shaped by relationships, structural inequalities and the wider institutional environment in which care is delivered. The difficulty lies not in any deficiency in women’s decision-making, but in a model of autonomy that presumes independence from these constraints. As a result, the legal test is ill-fitting—not because the principle of autonomy is flawed, but because its translation into practice relies on an unduly narrow conception of decision-making that abstracts choice from the conditions in which it is formed.

This narrowness is reinforced by gendered stereotypes that diminish women’s voices in clinical encounters. Women are often characterized as overly emotional, unstable, or incapable of rational decision-making. As Bell and others observe, gendered identities are constructed linguistically: women are diminished as ‘emotional’ or ‘sensitive’ while male authority is bolstered by terms such as ‘order’ and ‘management’.^105^ These stereotypes legitimize paternalism in maternity care, permitting healthcare professionals to substitute their own judgment for women’s expressed preferences under the guise of protection. Informed consent then risks becoming a one-sided dialogue in which women’s accounts of their fears, hopes, and values are marginalized or dismissed.

Beyond stereotypes, structural gender inequities also undermine women’s ability to exercise autonomy. Women are disproportionately affected by poverty and socioeconomic disadvantage, often carrying non-negotiable caring responsibilities alongside limited access to healthcare resources.^106^ In practice, family decisions are shaped by power imbalances and gender norms that undermine individual freedom of choice, for example, women sacrificing paid work opportunities or personal needs to prioritize children or partners. These dynamics have lasting financial and social consequences, with women faring worse than men on separation, and sometimes facing restricted access to medical care as a result.^107^ As Doyal argues, gender equity requires not identical treatment for men and women but attention to the different inputs necessary for human flourishing.^108^ Without recognition of these structural inequities, appeals to ‘equal treatment’ or ‘individual choice’ will inevitably fail women.

These gendered stereotypes and structural inequities illustrate the limitations of an individualistic account of autonomy in maternity care. It overlooks the realities of women’s lives, including their social obligations, economic constraints and the emotional context of decision-making. A relational approach offers a more adequate framework because it is capable of engaging with the social, institutional and interpersonal conditions through which decisions are formed and expressed.^109^^,^^110^

These insights also show that the problem cannot be resolved by legal doctrine or ethical guidance alone. So long as autonomy is framed as an individualistic, procedural exercise, it fails to capture the relational, cultural and institutional dynamics that shape women’s experiences of pregnancy and childbirth.

For autonomy to have substantive meaning in maternity care, reform must move beyond procedural safeguards and abstract rights. It must address the cultural assumptions and institutional practices that silence women and perpetuate paternalism. Only by confronting both the surface-level failures identified in the inquiries and the deeper epistemic foundations that sustain them can meaningful, patient-centred autonomy be realized.

### II. Why relational autonomy could work in this context

What is required is a model that genuinely accommodates women’s questions, perspectives, and preferences; one that situates choice within the broader social, emotional, and institutional realities of pregnancy. This section sets out the foundations of such a model: relational autonomy.

The starting point is to reject a conception of autonomy constrained by deference to medical opinion. Instead of treating patient choice as something that can be overridden in the name of clinical judgement, maternity care requires a framework that supports meaningful dialogue and respects the lived experiences of pregnant women. Rational decision-making is not always immediate or purely objective; in contexts of vulnerability and complexity, pregnant women need time, support and partnership to make choices that reflect their values and priorities.

This position aligns with established bioethical insights. Beauchamp and Childress emphasize that autonomy depends upon the presence of conditions that foster rational agency,^111^ while scholars such as Coggon and Miola highlight the distinctions between autonomy and liberty and the limits of assuming a detached, consistently rational patient.^112^ These insights suggest that autonomy cannot be reduced to a box-ticking exercise; it must be relational, context-sensitive and embedded in trust and communication.

The case law makes clear that this is not an abstract debate but can be a matter of life and death. In *Montgomery*, Mrs Montgomery was denied the opportunity to choose a caesarean section because her clinician assumed she should undergo a vaginal delivery. Had her autonomy been respected, her child’s injuries might have been avoided. In *McCulloch*, the failure to meaningfully explore alternatives meant that the patient was not told about the option of NSAIDs, a treatment that could have saved his life. In both cases, the courts were required to consider the relationship between professional judgement and patient choice. While the clinical circumstances differed significantly, both cases illustrate the importance of ensuring that patients are able to engage meaningfully with treatment decisions and available alternatives. These examples illustrate a powerful truth: autonomy is itself a clinical safeguard. It is not merely a legal or ethical formality but a vital mechanism through which harm is prevented, and lives are preserved. In maternity care especially, respecting women’s autonomy is essential not only to dignity but to safety.

Relational autonomy provides the framework for this shift. By moving beyond individualism, it reconceives decision-making as shaped by relationships, communication and power. It recognizes that patients are not isolated rational actors but social beings whose agency is influenced by trust in professionals, the availability of support networks and the institutional cultures within which choices are made.^113^ In maternity care, at least four interrelated features make relational autonomy particularly apt.

First, relational autonomy foregrounds communication and dialogue as the foundation of choice. Autonomy is sustained through meaningful engagement, not one-sided disclosure.^114^ This mirrors the GMC’s consent guidance, which repeatedly emphasizes supported decision-making, partnership and the need for clinicians to explore what matters to patients. However, the maternity inquiries demonstrate a persistent gap between this ethical ideal and clinical reality. Consent was routinely reduced to a procedural formality, with women ‘consented’ rather than genuinely involved in decisions about their care. A relational approach demands active listening, genuine discussion of alternatives and respect for women’s values and concerns. Autonomy cannot flourish where voices are silenced.

Second, relational autonomy explicitly attends to trust and power. Trust is central to the clinician-patient relationship, but it cannot be demanded; it must be earned through openness, honesty and respect. Relational autonomy requires recognition of the power imbalances that characterize maternity care, where clinicians hold authority not only through expertise but through institutional position.^115^ A relational model challenges the ‘doctor knows best’ mentality by requiring decision-making to be shared rather than substituted. Relational autonomy thus responds not only to power asymmetries but also to the epistemic injustice embedded in clinical decision-making, by re-centring patients as credible knowers of their own experiences.

Third, relational autonomy insists that context and support are not external to autonomy but conditions of it. Pregnant patients’ decisions are shaped by social, emotional and practical realities: family responsibilities, cultural expectations, financial insecurity, language barriers, and prior experiences of trauma or disrespect in healthcare.^116^ An individualistic model tends to treat these factors as irrelevant to rational agency.^117^ Relational autonomy recognizes that without adequate support, including time, clear explanations, continuity of care, and accessible information, choice is formally offered but not meaningfully available.^118^

Fourth, relational autonomy takes vulnerability seriously without turning it into a justification for control. Pregnancy is a period of heightened physical and emotional vulnerability, and maternity care often involves pain, fear, urgency and uncertainty.^119^ Rather than using vulnerability to legitimize paternalism, a relational model treats vulnerability as a common feature of human life that calls for compassion, support and respectful partnership. This includes rejecting stereotypes of irrationality and ‘baby brain’ and affirming women’s capacity for agency within their lived circumstances.^120^

These core tenets show why relational autonomy is not simply an attractive ethical ideal but a framework that directly targets the mechanisms through which autonomy is routinely undermined in maternity care: silence, hierarchy, lack of support and the mismanagement of vulnerability. The constructive development of this framework, and its translation into practice through training, guidance and institutional reform, is the subject of the companion paper. For autonomy to be meaningful rather than illusory, maternity care must move beyond the narrow confines of an individualistic understanding of autonomy.

While relational autonomy offers a more context-sensitive framework, it is not without risks. By foregrounding vulnerability, dependency and social context, it may inadvertently provide a basis for renewed paternalism. In particular, clinicians may interpret a patient’s relational circumstances or emotional state as evidence of compromised agency, thereby justifying intervention in the name of protection. In maternity care, where assumptions about vulnerability and maternal responsibility are already deeply embedded, this risk is especially acute. A relational framework must therefore be carefully constrained: attention to context must not become a mechanism for overriding patient choice, but a means of supporting it. Without such constraint, relational autonomy risks replicating the very paternalism it seeks to challenge.

## E. Conclusion: towards relational autonomy in maternity care?

Autonomy in maternity care must be reconceptualized. The failures documented demonstrate that autonomy in maternity care is undermined in two distinct but related ways: it is frequently not engaged at all in practice, and where it is invoked, it operates through a model that is conceptually incapable of addressing the relational, institutional and power-laden conditions in which decisions are made. The individualistic model that underpins current legal and ethical frameworks has, in practice, been translated into a focus on procedural disclosure and risk communication. In this operationalized form, it has proved inadequate and, at times, actively harmful, silencing women, obscuring their preferences, and legitimizing paternalism under the guise of informed consent.

The failures exposed are not isolated lapses but features of a wider institutional culture shaped by gendered assumptions, epistemic injustice, and entrenched clinical hierarchies. Women are frequently treated as less competent, more emotional, and less entitled to full participation in decisions about their care, particularly when their choices diverge from clinical expectations or prioritize values beyond biomedical outcomes. These cultural norms undermine the implementation of legal standards such as those articulated in *Montgomery*, and they are exacerbated by recent developments such as *McCulloch*, which risks reinstating professional gatekeeping over what information women should receive.

Relational autonomy may offer a more realistic and ethically grounded alternative. By situating decision-making within the relational conditions that make autonomy possible—trust, communication, support, and attention to structural vulnerability—it addresses precisely the failings identified in maternity services. Respect for autonomy thus becomes more than a matter of legal compliance; it functions as a safeguard against harm, a means of restoring trust, and a foundation for genuinely patient-centred care.

In practical terms, this paper does not call for a wholesale reform of the legal test or the creation of new regulatory duties. Rather, its contribution is to highlight the persistent gap between legal and ethical guidance and the lived reality of pregnant women in the UK, and to argue that bridging this gap may require a shift towards a relational framework for maternity care. This is both a cultural and institutional task, one that demands changes in communication, professional norms, and organizational priorities if autonomy is to function as a meaningful rather than theoretical right.

Nowhere is this need more urgent than in maternity care, where MBRRACE-UK and successive inquiries reveal the tragic consequences of failing to hear and value women’s voices. Until legal, ethical, and clinical systems align to make listening a substantive institutional priority, rather than a procedural formality, autonomy will remain constrained, conditional, and too often illusory.

The companion article will develop this argument further by providing the practical framework required to embed relational autonomy in maternity care, outlining what reform might look like in concrete clinical and institutional settings.

## Footnotes

1 G Thomson and others, ‘Minoritised Ethnic Women’s Experiences of Inequities and Professional/System Failures in UK Maternity Services’ (2022) 22 *BMC Pregnancy and Childbirth*, 958; A O’Dowd, 'Maternity Care: National Failures—Not Local Issues—Are to Blame for Poor Care, Patient Safety Body Warns' (2025) 390 BMJ.

2 O' Dowd A. Maternity care: national failures—not local issues—are to blame for poor care, patient safety body warns BMJ 2025; 390:r1781

3 G Thomson and M-C Balaam, ‘Researchers’ Perspectives of Self-Agency within a Context of Violence and Harm in Maternity Care’ (2024) 134 *Midwifery*; H Rayment-Jones and others, ‘Project20: Maternity Care Mechanisms that Improve (or Exacerbate) Health Inequalities for Pregnant Women with Social Risk Factors’ (2023) 36 *Women and Birth*.

4 Gill Thomson, Marie-Clare Balaam, ‘Researchers’ perspectives of self-agency within a context of violence and harm in maternity care (2024) 137 Midwifery 104120; Rayment-Jones H, Harris J, Harden A ... Project20: Maternity care mechanisms that improve (or exacerbate) health inequalities. A realist evaluation Women and Birth, 2022; 36, e314-e327.

5 L Abiola and others, ‘Barriers to Informed Consent in Obstetric Care During Childbirth from Practitioner’s Perspective: A Qualitative Study’ (2025) 15 BMJ Open e101591.

6 H Rayment-Jones et al, ‘How Do Women with Social Risk Factors Experience United Kingdom Maternity Care? A Realist Synthesis’ (2019) 46 Birth 461; SA Silverio and others, ‘Inside the “Imperfect Mosaic”: Minority Ethnic Women’s Qualitative Experiences of Race and Ethnicity During Pregnancy, Childbirth, and Maternity Care in the United Kingdom’ (2023) 23 BMC Public Health 2555.; MBRRACE-UK, *Saving Lives, Improving Mothers’ Care* 2024*: Lessons learned to inform maternity care from the UK and Ireland Confidential Enquiries into Maternal Deaths and Morbidity 2020–22*, National Perinatal Epidemiology Unit, October 2024.

7
*Montgomery v Lanarkshire Health Board* [2015] UKSC 11, [2015] AC 1430; AM Farrell and M Brazier, ‘Not So New Directions in the Law of Consent? Examining Montgomery v Lanarkshire Health Board’ (2016) 42 Journal of Medical Ethics 85; R Heywood, ‘RIP Sidaway: Patient-Oriented Disclosure—A Standard Worth Waiting For’ (2015) 23 Medical Law Review 455; N Harrison et al, ‘How *Montgomery* Is Reconfiguring Consent in the UK’ (2018) 392 The Lancet 102.

8 M Dalmaijer and others, ‘Cultural Competence of Maternity Care Professionals Caring for Majority Population Women with a Low Socioeconomic Position: A Scoping Review’ (2025) 22 Discover Public Health 93.

9 M Dixon-Woods and others, ‘Culture and Behaviour in the English National Health Service: Overview of Lessons from a Large Multimethod Study’ (2014) 23 BMJ Quality & Safety 106; S Batram-Zantvoort and others, ‘A Critical Review: Developing a Birth Integrity Framework for Epidemiological Studies Through Meta-Ethnography’ (2023) 23 BMC Women’s Health 530.

10 B Kirkup, *The Report of the Morecambe Bay Investigation*, The Stationery Office, March 2015.

11 B Kirkup, *Reading the Signals: Maternity and Neonatal Services in East Kent—the Report of the Independent Investigation* (HC 681, October 2022).

12 D Ockenden, *Findings, Conclusions and Essential Actions from the Independent Review of Maternity Services at the Shrewsbury and Telford Hospital NHS Trust* (Final Report, March 2022, HC1219).

13 D Ockenden, Terms of reference: Independent Maternity Review—Nottingham University Hospitals NHS Trust: Terms of Reference, Version 3, July 2025.

14 Press Release, 14 NHS trusts the focus of national maternity investigation, 15 September 2025, <https://www.gov.uk/government/news/14-nhs-trusts-the-focus-of-national-maternity-investigation> accessed 15 December 2025.

15
*Montgomery v Lanarkshire Health Board* [2015] UKSC 11, [2015] AC 1430 (SC).

16 J Miola, ‘On the Materiality of Risk: Paper Tigers and Panaceas’ (2009) 17 Medical Law Review 76; R Heywood and J Miola, ‘The Changing Face of Pre-Operative Medical Disclosure: Placing the Patient at the Heart of the Matter’ (2017) 133 Law Quarterly Review 296.

17
*McCulloch and others v Forth Valley Health Board* [2023] UKSC 26, [2023] 1 WLR 2286 (SC).

18 C Hobson, ‘Still No More Bolam, Please: McCulloch and others v Forth Valley Health Board’ (2024) 87 Modern Law Review 1586.

19 TL Beauchamp and JF Childress, *Principles of Biomedical Ethics* (8th edn, OUP 2019).

20 J Herring and P-L Chau, ‘My Body, Your Body, Our Bodies’ (2007) 15 Medical Law Review 34.

21 O O’Neill, ‘Some Limits of Informed Consent’ (2003) 29 Journal of Medical Ethics 4.

22 D O’Connor, *A Relational Approach to Medical Decision-Making and Best Interests: Autonomy, Capacity, and Consent from a Feminist Perspective* (PhD thesis, Cardiff University, 2023) <https://orca.cardiff.ac.uk/id/eprint/169145/1/2024O%27ConnorDPhD.pdf> accessed 27 August 2025.

23 J Nedelsky, *Law’s Relations: A Relational Theory of Self, Autonomy and Law* (OUP 2012) 4.

24
*Montgomery v Lanarkshire Health Board* [2015] UKSC [10].

25 ibid [11], [6].

26 ibid [7].

27 ibid [9] & [11].

28 ibid [8].

29 ibid [9].

30
*Sidaway v Board of Governors of the Bethlem Royal Hospital* [1985] AC 871; *Bolam v Friern Hospital Management Committee* [1957] 1 WLR 582; *Bolitho v City and Hackney Health Authority* [1998] AC 232.

31 ibid n22 at [28].

32 ibid [29].

33 ibid [74–80].

34 ibid [81–85].

35 ibid [86–91].

36 ibid [94], [101], [103].

37 ibid [104].

38 ibid [109–115].

39 ibid [115].

40 ibid & [116].

41 ibid.

42 L O’Donovan and others, ‘Medical Misogyny: Understanding Epistemic Injustice to Achieve Safer Healthcare for Women in the UK’ (2026) 34 Medical Law Review fwag009.

43 M Brazier, ‘Do No Harm—Do Patients Have Responsibilities Too?’ (2006) 65 Cambridge Law Journal 397; M Donnelly, ‘Best Interests, Patient Participation and the Mental Capacity Act 2005’ (2009) 17 Medical Law Review 1.

44 M Hevia and A Constantin, ‘Gendered Power Relations and Informed Consent’ (2018) *Health and Human Rights Journal* 20(2), 197.

45 J McCarthy, ‘Principlism or Narrative Ethics: Must We Choose Between Them?’ (2003) *Medical Humanities* 29, 65.

46 Hobson (n 16).

47
*Duce v Worcestershire Acute Hospitals NHS Trust* [2018] EWCA Civ 1307.

48 Hobson (n 16).

49
*McCulloch v Forth Valley Health Board* [2023] UKSC 26, [55]–[61].

50 Hobson (n 16).

51 R Heywood and J Miola, ‘Back to the Future? Identifying an Appropriate Legal Approach to Judging Disclosure of Alternative Medical Treatments’ (2025) 36 King’s Law Journal 16.

52 JM Laing, ‘Delivering Informed Consent Post-Montgomery: Implications for Medical Practice and Professionalism’ (2017) 33 Tottel’s Journal of Professional Negligence 128.

53 See for instance, NHS England, *Personalised Care and support Planning Guidance, Guidance for Local Maternity Systems*, Version 1, March 2021, PAR423.

54 National Institute for Health and Care Excellence, *Shared Decision-Making* (NG197, 2021).

55 P Leahy-Warren and others, ‘Factors Influencing Women’s Perceptions of Choice and Control During Pregnancy and Birth: A Cross-Sectional Study’ (2021) 21 BMC Pregnancy and Childbirth 667.

56 E Cave, M Brazier and R Heywood, *Medicine, Patients and the Law* (7^th^ edn, CUP 2023) 169.

57 C Doherty and others, ‘The Consent Process: Enabling or Disabling Patients’ Active Participation?’ (2017) 21 Health 205.

58 General Medical Council, *Decision Making and Consent* (November 2020).

59 J Craigie and others, ‘Support for Decision-Making Guidance in England: A Pragmatic Review’ (2025) 33 Medical Law Review.

60 Jillian Craigie, Antonia Alley, Maria Teresa Cotrufo, Michael Bach, Jodie Rawles, Isabel C H Clare, Matt Matravers, Francesca Happé, ‘Support for decision-making guidance in England: a pragmatic review’ (Summer 2025) 33 Medical Law Review fwaf021.

61 G Parker and others, *‘*Transcript: The Mess in the NHS’, *Financial Times* (London, 22 August 2025) <https://www.ft.com/content/deb47be4-32e4-4e47-a7a8-9528f6406f24> accessed 15 December 2025; D Campbell, ‘Hospital Patients Dying Undiscovered in Corridors, Report on NHS Reveals’ *The Guardian*, 16 January 2025 <https://www.theguardian.com/society/2025/jan/16/hospital-patients-corridors-royal-college-of-nursing-report-nhs> accessed 15 December 2025.

62 R Gilbar and J Miola, ‘One Size Fits All? On Patient Autonomy, Medical Decision-Making, and the Impact of Culture’ (2015) 23 Medical Law Review 375; ES Dove and others, ‘Beyond Individualism: Is There a Place for Relational Autonomy in Clinical Practice and Research?’ (2017) 12 Clinical Ethics 150; J Coggon and J Miola, ‘Autonomy, Liberty and Medical Decision-Making’ (2011) 70 Cambridge Law Journal 523.

63 Abiola and others (n 3); Rayment-Jones and others (n 4).

64 C Yuill and others, ‘Women’s Experiences of Decision-Making and Informed Choice about Pregnancy and Birth Care: A Systematic Review and Meta-Synthesis of Qualitative Research’ (2020) 20 BMC Pregnancy and Childbirth 343.

65 E Bell and others, ‘Time’s Up! Feminist Theory and Activism Meets Organization Studies’ (2018) 72 Human Relations 4; M Fricker, *Epistemic Injustice: Power and the Ethics of Knowing* (OUP 2007).

66 Nedelsky (n 21); N Stoljar and C Mackenzie, ‘Relational Autonomy in Feminist Bioethics’ in WA Rogers and others (eds), *The Routledge Handbook of Feminist Bioethics* (Routledge 2023**)** 21–24.

67 F Baylis, NP Kenny and S Sherwin, ‘A Relational Account of Public Health Ethics’ (2008) 1 Public Health Ethics 196; Nedelsky (n 21).

68 Kirkup (n 9) at 50.

69 Ockenden (n 10).

70 J Coggon, ‘Varied and Principled Understandings of Autonomy in English Law: Justifiable Inconsistency or Blinkered Moralism?’ (2007) 15 Healthcare Analysis 235.

71 Kirkup (n 9) at 4.

72 ibid.

73 ibid at 14.

74 Ockenden (n 10) at 139.

75 Kirkup (n 9) at 5.

76 Kirkup (n 8) at 4.

77 Kirkup (n 9) at 3.

78 ibid.

79 ibid at 9.

80 Kirkup (n 9) at 10.

81 ibid.

82 O’Donovan and others (n 41).

83 S Villarmea and B Kelly, ‘Barriers to Establishing Shared Decision-Making in Childbirth: Unveiling Epistemic Stereotypes about Women in Labour’ (2020) 26 Journal of Evaluation in Clinical Practice 515; MB Vela and others, ‘Eliminating Explicit and Implicit Biases in Health Care: Evidence and Research Needs’ (2022) 43 Annual Review of Public Health 477.

84 R Crawley, S Grant and K Hinshaw, ‘Cognitive Changes in Pregnancy: Mild Decline or Societal Stereotype?’ (2008) 22 Applied Cognitive Psychology 1142.

85 P Choi and others, ‘Supermum, Superwife, Supereverything: Performing Femininity in the Transition to Motherhood’ (2005) 23 Journal of Reproductive and Infant Psychology 167.

86 MR Hebl and others, ‘Hostile and Benevolent Reactions toward Pregnant Women: Complementary Interpersonal Punishments and Rewards That Maintain Traditional Roles’ (2007) 92 Journal of Applied Psychology 1499.

87 Fricker (n 63); Thomson and others (n 2); G Thomson and others, ‘Minoritised Ethnic Women’s Experiences of Inequities and Discrimination in Maternity Services in North-West England: A Mixed-Methods Study’ (2022) 22 BMC Pregnancy and Childbirth 958.

88 JR Abrams, ‘The Illusion of Autonomy in Women’s Medical Decision-Making’ (2014) 42 Florida State Law Review 17.

89
*Re MB (Caesarean Section)* [1997] 2 FLR 426.

90
*St George’s Healthcare NHS Trust v S* [1998] 3 All ER 673 (CA).

91
*Paton v British Pregnancy Advisory Service Trustees* [1979] QB 276; AV Hulme, ‘Anticipatory Declarations in Obstetric Care: A Relational and Spatial Examination of Patient Empowerment, Institutional Impacts and Temporal Challenges’ (2024) 32 Medical Law Review 530.

92
*Vo v France* 53924/00 [2004] ECHR 326.

93 S Wood-Goldbeck, ‘Women’s Autonomy in Childbirth’ (1997) 315 British Medical Journal 488.

94 GJ Annas, ‘Protecting the Liberty of Pregnant Patients’ (1987) 316 New England Journal of Medicine 1213.

95 H Cahill, ‘An Orwellian Scenario: Court-Ordered Caesarean Section and Women’s Autonomy’ (1999) 6 Nursing Ethics 494.

96 Yuill and others (n 62).

97 Z Khan and others, ‘Consent in Pregnancy: A Qualitative Study of the Experiences of Ethnic Minority Women’ (2024) 123 Patient Education and Counselling 108234.

98 J MacLellan and others, ‘Black, Asian and Minority Ethnic Women’s Experiences of Maternity Services in the UK: A Qualitative Evidence Synthesis’ (2022) 78 Journal of Advanced Nursing 2175.

99 Rayment-Jones and others (n 4).

100 MBRRACE-UK (n 4).

101 ibid.

102 The Equality and Human Rights Commission, *Pregnancy and maternity discrimination research findings*, 25 May 2018 <https://www.equalityhumanrights.com/guidance/business/pregnancy-and-maternity-discrimination-research-findings> accessed 16 December 2025.

103 Villarmea (n 82).

104 Coggon and Miola (n 60); Hulme (n 91).

105 Bell (n 63).

106 J Sims and M Butler, ‘Health and Environment: Moving Beyond Conventional Paradigms’ in G Sen, A George and P Östlin (eds), Engendering International Health: The Challenge of Equity (MIT Press 2002) 195.

107 A Heenan, ‘Neoliberalism, Family Law, and the Devaluation of Care’ (2021) 48 Journal of Law and Society 386; H Fisher and H Low, ‘Recovery from Divorce: Comparing High- and Low-Income Couples’ (2016) 30 International Journal of Law, Policy and the Family 338.

108 L Doyal and I Gough, *A Theory of Human Need* (Macmillan Education, 1991) 59–69, 155–161; MC Nussbaum and J Glover, *Women, Culture and Development: A Study of Human Capabilities* (OUP 1996).

109 J Craigie and others, ‘Support for Decision-Making Guidance in England: A Pragmatic Review’ (2025) 33 Medical Law Review.

110 Craigie et al, (n 59).

111 Beauchamp and Childress (n 17).

112 Coggon and Miola (n 60).

113 J Christman, ‘Relational Autonomy, Liberal Individualism and the Social Construction of Selves’ (2004) 117 Philosophy Studies 143.

114 Craigie and others (n 109); J Kors and others, ‘Autonomy-supportive Decision-Making in Maternity Care during Prenatal Consultations: A Qualitative Interaction Analysis’ (2022) 12 *BMJ Open* e063463.

115 N Stoljar and C Mackenzie, ‘Relational Autonomy in Feminist Bioethics’ in WA Rogers and others (eds), The Routledge Handbook of Feminist Bioethics (Routledge 2023); O’Connor (n 20).

116 Rayment-Jones and others (n 4).

117 JK Walter and LF Ross, ‘Relational Autonomy: Moving Beyond the Limits of Isolated Individualism’ (2014) 133 Pediatrics 16.

118 Mackenzie and Stoljar (n 114).

119 Kors and others (n 113).

120 Hebl and others (n 85).

